# Supplementary material for: A User-Friendly Approach for Routine Histopathological and Morphometric Analysis of Skeletal Muscle Using CellProfiler Software
Source: Diagnostics (Basel). 2022 Feb 22;12(3):561. doi: 10.3390/diagnostics12030561 (PMC8947111; doi:10.3390/diagnostics12030561)
Supplement: Supplementary file 1 [file diagnostics-12-00561-s001.zip › Troubleshooting_guide.pdf]

Troubleshooting guide

Image analysis is a process made of several steps, which starts with image acquisition and ends with data analysis and visualization. During these steps, many errors or pitfalls can occur even to more expert researchers. With the following troubleshooting guide, we wanted to facilitate the use of MyoProfiler and SiriusProfiler pipelines for untrained or inexpert users. In the left part of the table are listed the problems that can occur during the steps of the pipeline, whereas in the right part is described the possible solution that can be accomplished to overcome the problem. Obviously, several errors can occur also in sample preparation, embedding, fixation, staining and acquisition, as already widely described in literature. Herein we will focus only to troubleshoot in our CellProfiler-based pipelines.

PROBLEM DETECTED	POSSIBLE SOLUTION
**DATASET IS NOT PRESENT OR CANNOT BE RUN**	This kind of error can happen when an image set or complete data set is not uploaded in Images module.<br>If data have been correctly uploaded in the Images module, it is advised to use the button “Extract metadata” in the Metadata module, selecting from the drop-down menu the option “Extract from image file headers”. If the error persists, check in NamesAndTypes module, making sure that the criteria used for file recognition and subdivision are properly assigned according to dataset name and structure.
**LAMIN SIGNAL IS TOO SHARP OR TOO BLURRY**	It can be related to module #06 Gaussian Filter. Acting on the increase or on the reduction of the sigma value could increase/reduce the sharpness of the images.
**INTERRUPTED LAMININ SIGNAL OR WITH HOLES IN BETWEEN SPACES**	In module #13 (Closing) it is possible to increase or decrease the “Size” value to fill or reduce the laminin respectively.
**BINARY LAMIN TOO NOISY, THICK OR BROKEN**	Module #15 (Threshold) can be adapted in order to have a better binary output of laminin images. Threshold can be adjusted with “Thresholding correction factor” option: between 0 and values <1 means that the

	correction factor is more lenient, 1 means no adjustment and >1 that it is more stringent.
**PARTIAL LAMININ THRESHOLDING**	If the image intensity is highly uneven, the use of an “adaptative” thresholding method instead of a “global” one, is advised. MyoProfiler_variant supplementary file has been made to overcome this problem.
**UNDER- OR OVERSEGMENTATION OF MUSCLE FIBERS**	In module #18 (IndentifyPrimaryObjects) is possible to manually fix the local maxima value. Selecting “No” in the option “Automatically calculate minimum allowed distance between local maxima?” is possible to adjust the clumping of the objects.
**LARGE OR SMALL FIBERS EXCLUDED FROM SEGMENTATION**	This problem could be related to module #18 (IdentifyPrimaryObjects) or to module #20 (FilterObjects). In module #18 the “Typical diameters of objects” can be adjusted and it corresponds to the Min and Max diameter (in pixels) of objects: this parameter can be measured empirically in input Laminin images. Moreover, in module #20 it is possible to filter objects depending on the area (in pixels).
**MUSCLE FIBERS FILTERED OUT OR ERROUNEOUSLY INCLUDED**	This problem can be fixed by defining minimum and maximum values for the measurement to filter in module #20 (FilterObjects): area, form factor and solidity.
**WRONG CONVERSION FROM PIXEL TO MICROMETERS**	Depending on the magnification used for image acquisition and on pixel/µm ration, the conversion factor has to be calculated for both modules #25 and #26 (CalculateMath). The conversion factor and µm value is calculated as follows: 1/(Pixel/µm).
**NUCLEI TOO NOISY, BLURRY OR SPOTTED**	In module #29 (MediumFilter) the salt-and-pepper background noise can be adjusted by setting an opportune pixel size of the dimension window; in module #30 (EnhanceOrSuppressFeatures) the speckle feature can be adjusted, depending on the typical size of nuclei.
**NUCLEI NOT CORRECTLY SEGMENTED**	Module #31 (IdentifyPrimaryObjects) can be adapted in order to have a better binary output of DAPI images. Threshold can be

	adjusted with "Thresholding correction factor" option: between 0 and values <1 means that the correction factor is more lenient, 1 means no adjustment and >1 that it is more stringent.
**NUCLEI ARE TOO MUCH SEGMENTED OR CLUMPED**	Together with a threshold adjustment, it is possible to manually fix the local maxima value in module #31 (IndentifyPrimaryObjects). Selecting "No" in the option "Automatically calculate minimum allowed distance between local maxima?" is possible to adjust the clumping of the objects.
**NUCLEI ERROUNEOUSLY INCLUDED OR EXLUDED**	In module #31 (IndentifyPrimaryObjects) the "Typical diameters of objects" to be be segmented can be adjusted. It is recommended to measure the typical Min and Max diameter value (in pixels) of nuclei.
**MUSCLE FIBERS NOT PROPERLY SHRUNK**	In module #33 (ExpandOrShrinkObjects) is possible to adjust the shrinking of muscle fibers by an appropriate pixel value. It is better to determine this value empirically.
**MACROPHAGES TOO NOISY, BLURRY OR SPOTTED**	In module #41 (MediumFilter) the salt-and-pepper background noise can be adjusted by setting an opportune pixel size of the dimension window; in module #42 (EnhanceOrSuppressFeatures) the speckle feature can be adjusted, depending on the typical size of nuclei.
**MACROPHAGES NOT CORRECTLY SEGMENTED**	Module #43 (IdentifyPrimaryObjects) can be adapted in order to have a better binary output of F4/80 images. Threshold can be adjusted with "Thresholding correction factor" option: between 0 and values <1 means that the correction factor is more lenient, 1 means no adjustment and >1 that it is more stringent.
**MACROPHAGES ARE TOO MUCH SEGMENTED OR CLUMPED**	Together with a threshold adjustment, it is possible to manually fix the local maxima value in module #43 (IndentifyPrimaryObjects). Selecting "No" in the option "Automatically calculate minimum allowed distance between local maxima?" is possible to adjust the clumping of the objects.

**NUCLEI NOT PROPERLY EXPANDED**	In module #44 (ExpandOrShrinkObjects) is possible to adjust the expansion of nuclei if needed.
**IMAGES NOT SAVED, SAVED IN THE WRONG PATH OR WITH THE WRONG SUFFIX**	In modules #27, #39 and #48 (SaveImages) is possible to select the path where in which to save the images and the suffix to add to each image name. In order to save the image, this module has to be ticked/enabled accordingly.
**OUTPUT SAVED IN THE WRONG PATH**	In CellProfiler, in "files"->"Preferences…" is possible to select the default input and output folders to be used for analysis.
**SLOW IMAGE PROCESSING**	In CellProfiler, in "File"->"Preferences" is possible to adjust the number of "workers" to be used. This number depends by the CPU used (i.e an i9 can use 14 workers). Furthermore, it is also recommended to limit the use of computer during image analysis and to close all module displays (Hide All Windows On Run).
**MEASUREMENTS MISSING FROM OUTPUT**	In the last module (ExportToSpreadsheet) is possible to select the measurements that we want to export in csv. Furthermore, it is possible to select path, prefix, and other settings for the output file/s. It is recommended to always check that all appropriate selections have been made properly.

Scientific Community Image Forum link: https://forum.image.sc
